# Assessment of a course of realistic surgical training during medical education as a tool for pre-residential surgical training

**DOI:** 10.1186/s12909-016-0568-6

**Published:** 2016-02-03

**Authors:** Dominik S. Schoeb, Eva Brennecke, Anne Andert, Jochen Grommes, Klaus T. von Trotha, Andreas Prescher, Ulf P. Neumann, Marcel Binnebösel

**Affiliations:** Department of General, Visceral and Transplantation Surgery, RWTH Aachen University Hospital, Pauwelsstrasse 30, 52074 Aachen, Germany; Department of Vascular and Endovascular Surgery, RWTH Aachen University Hospital, Aachen, Germany; Institute of Molecular and Cellular Anatomy, RWTH Aachen University, Aachen, Germany

**Keywords:** Medical education, Teaching, Academic acquisition, Manual skills, Practical education, Surgical education

## Abstract

**Background:**

In recent years the focus on practical skills in the German curriculum of medical school has increased greatly. In this study we evaluate the value of a practical surgery course for medical students as a tool for surgical education, as a way of enhancing interest in surgical fields, and as a method of influencing medical students to subsequently choose a surgical career.

**Methods:**

The *“Feel like a surgeon”-*course is an optional practical surgery course in which topographical anatomy and realistic surgical training using fresh human cadavers are combined for medical students of the RWTH Aachen University. Between 2010 and 2015 every student completed a survey before starting and after completing our course, and in 2015 a follow-up was performed. Using a standardized questionnaire, course quality, learning success and impact on post-instructional career and choice of profession was evaluated.

**Results:**

In total, 82 students attended our course between 2010 and 2015 and took part in the evaluation. Evaluation of the course was positive overall, with an average grade of 1.4° ± °0.50. Significant improvement of basic, as well as more complex surgical skills and theoretical knowledge was noted. Furthermore, self-confidence for patient related assignments improved as well. In the follow-up evaluation, a high level of recommendation for surgical residents was seen, as was a high influence of the course on our students’ career choice, although no significant change in career plans before and after taking the course was noted.

**Conclusions:**

Our results indicate that a practical surgical course can be a valuable tool to prepare students for a surgical residency and to improve their practical skills generally.

**Electronic supplementary material:**

The online version of this article (doi:10.1186/s12909-016-0568-6) contains supplementary material, which is available to authorized users.

## Background

In recent years, the focus on practical skills in the German curriculum of medical school has increased greatly In order to improve the preparation of medical students for the tasks required of a physician on the floors, routinely needed practical skills are expected to be included in the topics taught to medical students. This was emphasized in the release of a new version of the German Licensing Regulations (ÄAppO) in 2002, which included substantial increases in the importance of teaching and examining practical medical skills [1]. The weighting of the oral practical examination at the end of medical school was subsequently increased and graded block placements were introduced. These conditions put pressure on the medical faculty to address the introduction of hands-on teaching content and to expand the systematic teaching and examination of practical skills [2]. In 2002 the medical faculty of the RWTH Aachen responded to these challenges by introducing a unique, radically changed curriculum where clinical and theoretical knowledge was taught as a whole entity from the beginning. In addition to these changes, new learning facilities were introduced as well, such as a Skill-Center for practicing routine medical skills on dummies.

In this environment, the Department of General, Visceral and Transplantation Surgery of the RWTH Aachen university also faced the additional challenge of the fading attractiveness of surgical fields for graduating medical students and the consequent lack of candidates for their surgical residencies [3, 4]. In response to this situation, the surgical department decided to restructure their teaching program by adding realistic surgical training to the medical student curriculum [5]. This *“Aachen model”* offers medical students realistic, practical surgical training through optional courses with consecutive curricula and block placements implemented into the medical school curriculum [5]. In this system, the surgical department offers students the opportunity to improve their technical surgical skills and knowledge base at every stage of their education. In doing so, technical skills are not only greatly improved, but the repetitive contact with the surgical field is intended to increase the number of well-prepared candidates for surgical residency positions. [5].

A key element of this practical surgical training for medical students in Aachen is an optional course named “*Feel like a surgeon”,* in which topographical anatomy and realistic surgical training are combined. Originally designed for surgical residents as a recapitulation of topographic anatomies, it soon became very popular with practical year students and was subsequently introduced as an optional course for medical students in 1997. At this time trauma and visceral surgery residents served as tutors for the class, and topics included general surgery and orthopedic surgery procedures. It was revised and restructured several times and has existed in its current form since 2008, when the orthopedic surgery portion was transferred to a separate, free standing course. It is specifically designed not only to teach hands on surgical skills and recapitulate macroscopic anatomy, but to explicitly prepare students for a surgical career while encouraging them to follow this path. Former studies have shown a need for the development of teaching tools to help medical students entering skills oriented fields like surgery. Here, the transition from theory to clinic is most difficult, and can result in nervousness and feelings of incompetence [6]. A lack of practical skills seems to be of particular concern to students in this context [6]. Former studies using similarly structured courses have demonstrated a post-course improvement of technical skills [7], however no similarly comprehensive surgical course has been evaluated thus far. Also, no data on the influence of practical surgical courses on the final choice of medical discipline has been reported.

In this study we evaluate the value of this course as a tool for surgical education and as a way of increasing interest in surgical fields, thereby influencing medical students to choose a surgical career.

## Methods

### *“Feel like a surgeon”* - Course design

The *“Feel like a surgeon”* course is an optional class for a maximum of 25 medical students of the RWTH Aachen. The course is available for students in an advanced stage of medical education. It is technically open for all eligible medical students, but owing to didactic reasons and due to high demand in the last 5 years, prerequisites include successful completion of macroscopic anatomy, as well as basic and advanced surgical skills courses, These classes are taught in the medical skills lab of the RWTH Aachen by surgical department faculty.

The model of surgical training has been described before [5]. Briefly, the basic skills course is an elective class for medical students in the penultimate year of medical education. It is held weekly and consists of knotting, education in surgical suture material and instruments, as well as simple suture techniques. The advanced skills course is an optional course following completion of the basic skill course. It contains a review of surgical suture and knotting techniques, as well as basic and advanced conventional and laparoscopic suture techniques [5].

The *“Feel like a surgeon”* course is planned to span over one semester and includes 3 h of teaching per week over two days, containing theoretical and practical content. The initial 3 weeks (9 h) are used for intensive review of basic and advanced surgical techniques as mentioned before. The following part of the course is comprised of two parts. Each week consists of a theoretical lecture, where the anatomy of a certain region is recapitulated followed by the presentation of a surgical procedure performed in this anatomical region. The following day, students then perform this surgery on a human cadaver. To make the experience as close to reality as possible, procedures are performed on fresh cadavers not yet fixed in formaldehyde whenever possible. On average, a majority of students work on the unpreserved cadavers. If not enough fresh material is available, rotation between cadavers is encouraged to enable all students to gain the most realistic experience. The cadavers are stored and prepared at the Institute of Molecular and Cellular Anatomy. All specimens are received from individuals who voluntarily donated their bodies to be used in medical research and education pre-mortem through the body donor program of the Institute of Anatomy at the Technical University Aachen (RWTH). During the practical session, 4 to 5 students operate on the same cadaver under the supervision of at least 1 surgical resident tutor. All necessary surgical instruments to perform the surgery are provided and the procedure is performed as it would be in a real surgical setting. The procedures include: open appendectomy, inguinal hernia repair (ventral suture and mesh repair), carotid and femoral thromboendarterectomy, open tracheotomy, chest tube insertion, thoracotomy, hemithyroidectomy, conventional gall bladder resection, retrocolic gastroenterostomy, small bowel resection with end-to-end anastomosis as well as ileocecal resection with side-to-side anastomosis. At the end of the course, students are educated in laparoscopic skills using a computer aided laparoscopy trainer (Simbionix LAP mentor®, Cleveland, Ohio, USA) as well as laparoscopic dummies (D-box®, Lapskill Medical, Oslo, Norway) in 4 sessions.

### Participant information and data collection

Between 2010 and 2015, a total of 82 students participated in the course. Since 2010, every student was asked to fill out a standardized, anonymous questionnaire before and after participating in the course. Written informed consent and approval of the ethics board of the university hospital of Freiburg was obtained. Participants were assured that all data would be treated anonymously and confidentially. Recorded demographic parameters included gender, age, semester in medical school and former status as a paramedic. In 2015, all participants since 2010 were given a follow-up questionnaire. Because the 2015 students had recently finished the course, no follow-up was performed on this group.

### Assessment of course impact on career choice and skill acquisition

As mentioned above, every student completed a survey before starting and after completing our course. A scale from 1 to 6 was used for rating each statement, where 1 stands for “no agreement at all” and 6 stands for “complete agreement”. In addition, the students graded the whole course using standard German grading system, where 1 equals “very good” and 6 equals “insufficient”. General assessed features of our questionnaire (Additional file 1) included: number of attended classes, time invested in medical school per week, self-assessed ease of acquiring practical skills, preference of learning practical skills on patients, and motivation to attend the course as a means to prepare for examination. All students were asked to evaluate the concept, sufficiency of time allotted and the overall quality of the classes. Course effectiveness in teaching practical abilities was self-assessed by the students pre- and post-instruction for skills such as performing a surgical knot, performing a single-knot or intracutaneous suture, identifying anatomical structures as well as feeling of self-confidence in performing practical procedures on a patient. The success of our course in terms of recruitment of surgical prospects was evaluated by recording of pre- and post-instructional career plans as well as final career choice per follow-up. Also in the follow-up, all students were asked to self-asses the value of the course for their post-instructional career in terms of theoretical knowledge, hands-on abilities during the practical year and as preparation for their surgical residency if they pursued a surgical career.

### Statistical analysis

Statistical analysis has been carried out using the Statistical Package for Social Sciences software (SPSS®, Version 20.0, Chicago, IL, USA). Differences between groups were analyzed by Kruskal-Wallis test for non-parametric data and in case of significant differences confirmed by Mann–Whitney test. For numeric data, differences were analyzed by ANOVA and in case of significance confirmed by *T*-Test. *P*-values < 0.05 were considered to be significant. All data are represented as mean ± standard deviation.

## Results

### Demographic information

In total, 82 students participated in our course between 2010 and 2015 with a gender distribution of 56 % (*n*° = °46) male and 44 % (*n*° = °36) female students. The age of the participants ranged from 20 to 31 years with an average of 23.4 years. The majority of students attended the 7^th^ or 8^th^semester of medical school (7.68° ± °1.57) with a range from 7 to 13 semesters. Among all participants, 9 (11 %) reported previous practical experience as paramedics while 5 students (6 %) reported previous experience in nursing. 83 % (*n*° = °68) of all participants had no previous practical experience in the medical field prior to medical school. (Table 1).

**Table 1:** Participant Information

	All Participants (*n = 82*)
Age(yrs)	23,68° ± °2,10 (20–31)
Gender(male/female)	46/36
Paramedic experience(y/n)	9
Nursing experience(y/n)	5
Study semester	7,68° ± °1,57 (7–13)

Age is given as median with range in years; y = yes, *n* = no

### Postinstructional student evaluation

**Fig. 1:**
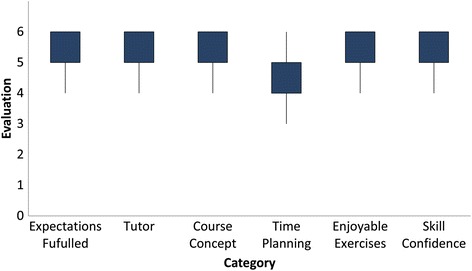
Student evaluation of course quality: On a scale from 1 to 6 students rated their level of satisfaction with time planning, motivational skills of course tutors, course concept and time schedule, the level of enjoyment when performing the exercises and the amount of confidence in their practical skills gained through this class. (1 stands for “no agreement at all” with the statement at hand and 6 stands for “complete agreement”)

Student feedback for our skills course was generally positive, with an overall grade of 1.4° ± °0.50 and a range from 1 to 2. Also, the overall grade significantly (*p*° = °0.004) improved over the years with an average grade of 1.8° ± °0.41 in 2011 to an average grade of 1.21° ± °0.43 in 2015. In addition, detailed aspects were rated positively as well. On a scale from 1 to 6, with 6 symbolizing “full agreement”, participants highly agreed to the class concept (5.00° ± °0.43), the defined learning goals (5.20° ± °0.79), the efficiency of the practical training (5.12° ± °0.43) and the motivation through the tutoring physicians (5.44° ± °0.43). Likewise, the quality of the anatomical specimens the students operated on was satisfactory (4.61° ± °1.18) and the practical training was rated efficient (5.12° ± °0.86) and engaging (5.63° ± °0.69). Time management (4.50° ± °1.30) was also generally agreed with. As a final point, the expectations of students were almost entirely met (5.23° ± °0.72) (Fig. 1).

### Learning success

**Fig 2:**
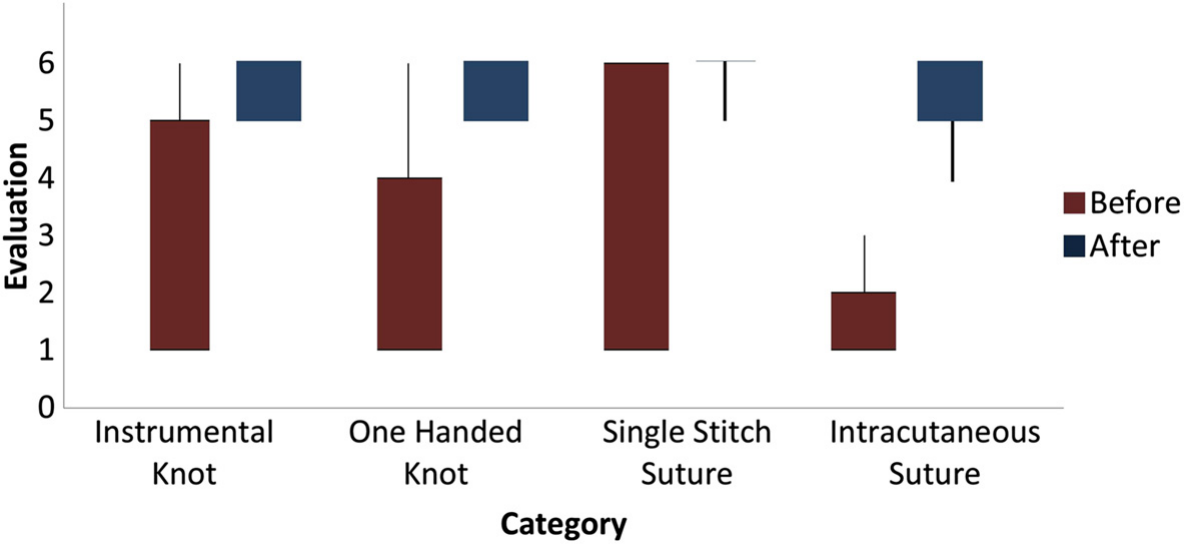
Student evaluation of course quality: On a scale from 1 to 6, students rated their level of confidence in the respective practical skills before and after finishing our practical class. For all examined skills a significant increase was noted. (1 stands for “no confidence in ability to perform this task” with the statement at hand and 6 stands for “full confidence in ability to perform this task”)

Training success was evaluated by self-evaluation before and after taking part in our skills course. Basic surgical skills as well as relevant anatomical knowledge were asked to be ranked on a scale from 1 to 6, with 6 symbolizing full confidence in the rated ability. Significant improvement was recorded for all evaluated skills, which were the ability to tie a surgical knot using instruments (*p* < 0.001) as well as to tie a one handed surgical knot (*p* < 0.001), the ability to suture using single stitches (*p* < 0.001) as well as performing an intracutaneous suture. More advanced skills trained in our class, like tying a surgical knot deep inside a body cavity, also showed significant improvement (*p* < 0.001). The self confidence in theoretical knowledge (*p* < 0.001) as well as in the identification of anatomical structures (*p* < 0.001) increased significantly. Ultimately, the sense of preparedness of all participants for working on actual patients improved significantly (*p* < 0.001) with an initial level of confidence of 3.01° ± °1.38 compared to a level of confidence of 4.72° ± °1.02 afterwards. In addition, the readiness to assist a trained surgeon in one of the taught procedures was 4.42° ± °1.04 afterwards. (Fig. 2).

### Follow-up evaluation and impact on participant career choice

Follow up was performed in May 2015. Median follow-up was 31 months ranging from 13 to 40 months. Former participants were asked to evaluate the class again with specific focus on its impact on their current medical practice and their career choice. Again a scale from 1 to 6, with 1 symbolizing “total disagreement” to 6 symbolizing “full agreement” with the discussed topic, was used. From the 82 participants of the original cohort, 67 were included in the follow-up, as 15 had just finished the course at the time of this evaluation. Of the 67 individuals included in the follow-up, 55 were already practicing as physicians, amongst which the rate of residents in general surgery or surgery related fields (urology, gynecology, orthopaedics, trauma surgery) was about 56 %. Generally, the course was rated positively in the follow-up as well, with a very high rate of recommendation for people going into a surgical residency (5.78° ± °0.47), although a general recommendation was not fully agreed with (3.58° ± °1.61). For the post instructional career of the participants, the course was largely rated as being generally advantageous (5.0° ± °1.04) and as improving technical skills (4.33° ± °1.43). No benefit was seen for behavior in the operation theatre (2.55° ± °1.46) or for handling of patients in general (2.26° ± °1.12), but practical skills (4.78° ± °1.04) and passing clinical clerkships (4.11° ± °1.51) were perceived as easier by having completed our skills course. Possible room for improvement was seen by the former participants in the potential addition of more education about surgical instruments (3.50° ± °1.46) and of a course handbook (4.09° ± °1.59). The impact on the career choice of the participants was rated as significant at a level of 4.31° ± °1.56 of agreement with the statement that this class solidified the participant’s choice of career. Also, the follow-up reflected that the decision for a surgical career was greatly affected by taking our course (4.26° ± °1.59), although candidates leaning towards a non-surgical career did usually not change their opinion (1.42° ± °1.11). Comparing the career plans before taking our class with afterwards showed an increase in the consideration of a residency in surgery (4.41° ± °1.52 vs. 4.72° ± °1.41) without however reaching any significance (*p* = 0.141). Also, no significant differences were seen when comparing the pre- and post-instructional career plans with the choice of career on follow up.

### Gender specific analysis

Overall satisfaction with our class showed no relevant differences between female and male participants. Overall grading was 1.38° ± °0.49 for female participants and 1.47° ± °0.50 for male participants (*p* = 0.446) and the level of recommendation showed no significant difference (*p* = 0.102). Also, learning success showed mostly similar results between female and male participants, with a tendency for higher self-confidence in their post-instructional practical skills in female participants. A significant difference was noted for complex surgical skills post-instruction, with female students showing a significantly higher confidence when tying a surgical knot inside a body cavity compared to male students (*p* = 0.042). No significant differences were found in the influence of the course on career choice or the career choices in general between female and male students (*p* = 0.568, *p* = 0.360).

## Discussion

As the focus of medical education begins to shift away from the classical form of transferring book-knowledge through teacher centered education, towards imparting practical skills necessary to be ready for real-life medical practice, the necessity for medical faculty to implement new teaching methods into the medical school curricula is imperative. In recent years, especially since the release of a new version of the German Licensing Regulations (ÄAppO) in 2002 [1], a trend towards the implementation of fully reformed medical school curricula as well as skill centers and practical courses in German medical schools has been seen. The surgical department in Aachen followed this trend as well by changing their teaching concept and starting a newly designed practical surgery course for medical students where practical and theoretical knowledge is taught through the active performance of real surgical procedures on anatomical specimens. To achieve an absolutely realistic situation, fresh human cadavers were used for the surgical procedures, thereby allowing students to learn topographic anatomy, preparation and tissue handling in as realistic a setting as possible. This is an outstanding feature of our course and therefore a notable advantage compared to courses using fixed human cadavers.

Our goal was to teach students practical surgical skills through a completely student centered teaching model and to increase learning success by combining the acquirement of new skills with the immediate real life application of these skills. This way, we wanted to achieve not only a very appealing learning atmosphere and a fun learning experience, but also to interest students early in their careers to pursue surgical fields. As these reported results show, we successfully executed a high quality course and were able to provide a teaching experience which a majority of students rated as enjoyable. This is especially important as an appealing learning environment directly leads to higher attendance rates [8] and higher long term teaching success. Our results furthermore indicate a significant improvement in basic surgical skills as well as more complex surgical skills and a readiness level to participate in real life surgeries. A clear limitation of this study is the application of a self-evaluation system to rate the learning success. To emphasize the validity of our results, a higher level of analysis as well as a control group is definitely desirable. However, studies have shown the validity of self-evaluation [9] to evaluate one’s practical skills when it comes to the application of surgical skills, because no objective methods are available. In order to further validate our results, we are planning a further evaluation for the upcoming courses using a standardized and timed skills test. Our current data however provides clear insight into the importance of practical skills courses. Furthermore, an important aspect is the consideration of the strong gain in self-confidence, which was clearly achieved by our course. Graduates of our class showed high levels of readiness for patient interaction and for the application of their gained skills. Also, a high level of preparedness for assisting in the trained procedures, as well as starting the clinical clerkships, was noticed. This is especially important, as a self-confident student doctor or physician is able to gain a patient’s trust more easily, reduce their stress level and ensure their compliance [10], especially when an invasive procedure is about to be performed. This is also underlined by our long-term follow-up, which showed a clear benefit of this skills class in furthering the practical education of the respondents. Respondents also strongly recommended that future surgical residents take the course.

However, the impact of our class on career choice and the decision for a surgical career is not as clear. Studies have shown previously that a lack of surgical mentorship can result in disinterest in surgical fields [11, 12]. The importance of early positive experiences in surgical fields has been particularly emphasized [13]. As a secondary goal of our course was to encourage student interest in surgical fields and to recruit talented individuals, we targeted students at the beginning of their 3^rd^ year in our 6 year medical school program and therefore at the very beginning of their clinical education. However, pre- and post-instructional evaluation showed no significant increase in the choice of a surgical career, although a follow-up evaluation rated the value of our class as influential in final career decision as relatively high. A possible explanation for the lack of significance might be a selection bias, as mainly students interested in surgery choose to attend our class. Furthermore, an associated selection might originate from the fact that students who did not successfully graduate from the basic and advanced skills course did not qualify to register for the *Feel Like a Surgeon* course. This is also emphasized by the high number (over 50 %) of course graduates who are practicing in surgical fields according to our follow-up.

As previous practical teaching models have been reported to show a different outcome depending on gender [14] and gender has been described as an influential factor on learning [15], a gender specific analysis was performed. Here, mostly similar results for confidence levels and learning success were reported. A small advantage in confidence gain for complex surgical skills in female students was noted, which is rated pleasantly by the authors of this study, as more and more female students enter the formerly male dominated field of surgery [16] and more female orientated teaching models are therefore desirable.

## Conclusion

Our results indicate that a practical surgical course can be a valuable tool to prepare students for a surgical residency and to improve their practical skills. Furthermore, it is a way to significantly improve students’ confidence in their practical skills and therefore improve their attitude towards patient related tasks. Our results also indicate that practical courses in the respective fields can be an important method for influencing and encouraging medical graduates in their choice for residency.

## Additional file

Additional file 1: Standardized questionnaire used for course evaluation and self assesment. (PDF 782 kb)

## Abbreviations

ÄAppO: Ärztliche Approbationsordnung
RWTH: Rheinisch Westfälisch Technische Hochschule
SSPS: Statistical Package for Social Sciences
