# Supplementary material for: Assessment of a course of realistic surgical training during medical education as a tool for pre-residential surgical training
Source: BMC Med Educ. 2016 Feb 3;16:45. doi: 10.1186/s12909-016-0568-6 (PMC4739082; doi:10.1186/s12909-016-0568-6)
Supplement: Additional file 1: — Standardized questionnaire used for course evaluation and self assesment. (PDF 782 kb) [file 12909_2016_568_MOESM1_ESM.pdf]

Semester: ____________ Date:________
First name:_____________ Last name:___________
Email:________________

1. Personal Information

1.1 Age

10er ☐ ☐ ☐ ☐ ☐ ☐ ☐ ☐ ☐ ☐
1er ☐ ☐ ☐ ☐ ☐ ☐ ☐ ☐ ☐ ☐
x0 x1 x2 x3 x4 x5 x6 x7 x8 x9

1.2 Gender ☐ male ☐ female

1.3 Previous experience ☐ nursing ☐ paramedic service

1.4 Current Semester

10er ☐ ☐ ☐ ☐ ☐ ☐ ☐ ☐ ☐ ☐
1er ☐ ☐ ☐ ☐ ☐ ☐ ☐ ☐ ☐ ☐
x0 x1 x2 x3 x4 x5 x6 x7 x8 x9

2. General questions

2.1 Number of attended classes:

10er ☐ ☐ ☐ ☐ ☐ ☐ ☐ ☐ ☐ ☐
1er ☐ ☐ ☐ ☐ ☐ ☐ ☐ ☐ ☐ ☐
x0 x1 x2 x3 x4 x5 x6 x7 x8 x9

2.2 Hours/week spent on studying for medical school (lincluding ectures, seminars, independent study time etc.):

10er ☐ ☐ ☐ ☐ ☐ ☐ ☐ ☐ ☐ ☐
1er ☐ ☐ ☐ ☐ ☐ ☐ ☐ ☐ ☐ ☐
x0 x1 x2 x3 x4 x5 x6 x7 x8 x9

2.3 I learned practical skills with ease so far. No agreement at all ☐ ☐ ☐ ☐ ☐ ☐ complete agreement

2.4 I prefer to train practical/clinical skills on patients. No agreement at all ☐ ☐ ☐ ☐ ☐ ☐ complete agreement

2.5 I use this class to prepare for exams. No agreement at all ☐ ☐ ☐ ☐ ☐ ☐ complete agreement

3. Class specific questions

3.1 How confident are you with tying a surgical knot? no confidence ☐ ☐ ☐ ☐ ☐ ☐ full confidence

3.2 How confident are you with performing both single handed surgical knots? no confidence ☐ ☐ ☐ ☐ ☐ ☐ full confidence

3.3 How confident are you with performing a single knot suture? no confidence ☐ ☐ ☐ ☐ ☐ ☐ full confidence

3.4 How confident are you with performing an intracutaneous suture? no confidence ☐ ☐ ☐ ☐ ☐ ☐ full confidence

3.5 How confident are you with performing a deep surgical knot? no confidence ☐ ☐ ☐ ☐ ☐ ☐ full confidence

3.6 How confident are you with your theoretical knowledge? no confidence ☐ ☐ ☐ ☐ ☐ ☐ full confidence

3.7 How confident are you with identifying anatomical structures? no confidence ☐ ☐ ☐ ☐ ☐ ☐ full confidence

3.8 How confident do you feel about the surgical part of your pracical year? no confidence ☐ ☐ ☐ ☐ ☐ ☐ full confidence

3.9 My final goal is a surgical residency no confidence ☐ ☐ ☐ ☐ ☐ ☐ full confidence

Semester: ____________ Date:________
First name:_____________ Last name:___________
Email:________________

4. General questions

4.1 The class fulfilled my expecations.	No agreement at all	☐	☐	☐	☐	☐	☐	complete agreement
4.2 I prefer to train practical/clinical skills on patients.	No agreement at all	☐	☐	☐	☐	☐	☐	complete agreement
4.3 The teachers motivated me to participate.	No agreement at all	☐	☐	☐	☐	☐	☐	complete agreement
4.4 The learning goals were clearly defined.	No agreement at all	☐	☐	☐	☐	☐	☐	complete agreement
4.5 The presenatation of the subjects were clearly understandable.	No agreement at all	☐	☐	☐	☐	☐	☐	complete agreement
4.6 The concept for the teaching of practical skills was recognizable.	No agreement at all	☐	☐	☐	☐	☐	☐	complete agreement
4.7 The pracice time was suffizient.	No agreement at all	☐	☐	☐	☐	☐	☐	complete agreement
4.8 The pracical exerzises were effecitve and helped me.	No agreement at all	☐	☐	☐	☐	☐	☐	complete agreement
4.9 The teachers often held monologues.	No agreement at all	☐	☐	☐	☐	☐	☐	complete agreement
4.10 The training on cadavers was fun.	No agreement at all	☐	☐	☐	☐	☐	☐	complete agreement
4.11 The participation in the class encouraged me to perform the trained procedures an a patient.	No agreement at all	☐	☐	☐	☐	☐	☐	complete agreement
4.10 The anatomical specimens used were of appropriate quality.	No agreement at all	☐	☐	☐	☐	☐	☐	complete agreement

5. Class specific questions

5.1 How confident are you with tying a surgical knot?	no confidence	☐	☐	☐	☐	☐	☐	full confidence
5.2 How confident are you with performing both single handed surgical knots?	no confidence	☐	☐	☐	☐	☐	☐	full confidence
5.3 How confident are you with performing a single knot suture?	no confidence	☐	☐	☐	☐	☐	☐	full confidence
5.4 How confident are you with performing an intracutaneous suture?	no confidence	☐	☐	☐	☐	☐	☐	full confidence
5.5 How confident are you with performing a deep surgical knot?	no confidence	☐	☐	☐	☐	☐	☐	full confidence
5.6 How confident are you with your theoretical knowledge?	no confidence	☐	☐	☐	☐	☐	☐	full confidence
5.7 How confident are you with identifying anatomical structures?	no confidence	☐	☐	☐	☐	☐	☐	full confidence
5.8 How confident do you fee about the surgical part of your practical year?	no confidence	☐	☐	☐	☐	☐	☐	full confidence
5.9 My final goal is a surgical residency.	no confidence	☐	☐	☐	☐	☐	☐	full confidence

Semester: ____________ Date:________ First name:_____________ Last name:_________ Email:________________

5. Class specific questions

5.10 How confident are you with assisting a trained procedure in an acual OR?

no confidence ☐ ☐ ☐ ☐ ☐ ☐ full confidence

6. Comments:

6.1 What part of the class did you like:

6.2 What part of the class did you dislike:

6.3 What suggestions for improvement do you have?

6.4 Which of the aspects did you like most:

☐ skill class ☐ general surgery ☐ trauma surgery

☐ neurosurgery ☐ laparoscopic surgery

7. Final grade for the class:

I give the total course the following grade:

☐ 1 ☐ 2 ☐ 3 ☐4 ☐5 ☐ 6
